# Long-term outcomes of adding alpha-glucosidase inhibitors in insulin-treated patients with type 2 diabetes

**DOI:** 10.1186/s12902-021-00690-0

**Published:** 2021-02-18

**Authors:** Fu-Shun Yen, James Cheng-Chung Wei, Mei-Chen Lin, Chih-Cheng Hsu, Chii-Min Hwu

**Affiliations:** 1 Dr. Yen’s Clinic, No. 15, Shanying Road, Gueishan District, Taoyuan, 33354 Taiwan; 2 Institute of Medicine, Chung Shan Medical University, No. 110, Sec. 1, Jianguo N. Rd., South District, Taichung City, 40201 Taiwan; 3 Department of Medicine, Chung Shan Medical University Hospital, No. 110, Sec. 1, Jianguo N. Rd., South District, Taichung City, 40201 Taiwan; 4 Graduate Institute of Integrated Medicine, China Medical University, No.91, Hsueh-Shih Road, Taichung, 40402 Taiwan; 5 Management Office for Health Data, China Medical University Hospital, 3F., No.373-2, Jianxing Road, Taichung, 40459 Taiwan; 6 College of Medicine, China Medical University, No. 110, Sec. 1, Jianguo N. Rd., South District, Taichung City, 40201 Taiwan; 7 Institute of Population Health Sciences, National Health Research Institutes, No.35, Keyan Rd., Zhunan Township, Miaoli 35053 Taiwan; 8 Department of Health Services Administration, China Medical University, No.91, Hsueh-Shih Road, Taichung, 40402 Taiwan; 9 Department of Family Medicine, Min-Sheng General Hospital, 168 ChingKuo Road, Taoyuan, 33044 Taiwan; 10 Faculty of Medicine, National Yang-Ming University School of Medicine, No. 201, Sec. 2 Shi-Pai Rd., Chung-Cheng Build. 11F Room 522, Taipei, 112 Taiwan; 11 Section of Endocrinology and Metabolism, Department of Medicine, Taipei Veterans General Hospital, No. 201, Sec. 2 Shi-Pai Rd., Chung-Cheng Build. 11F Room 522, Taipei, 112 Taiwan

**Keywords:** Insulin, Alpha-glucosidase inhibitors, All-cause mortality, Cardiovascular death, Coronary artery disease

## Abstract

**Background:**

In insulin-treated patients with type 2 diabetes mellitus (T2DM), glycemic control is usually suboptimal.

**Methods:**

This study compared the risks of mortality and cardiovascular events in insulin-treated patients adding or not adding alpha-glucosidase inhibitors (AGIs).

**Results:**

This cohort study included data from the Taiwan National Health Insurance Research Database. In total, 17,417 patients newly diagnosed as having T2DM and undergoing insulin therapy during 2000–2012 were enrolled. Overall incidence rates of all-cause mortality, hospitalized coronary artery disease (CAD), stroke, and heart failure were compared between 4165 AGI users and 4165 matched nonusers. The incidence rates of all-cause mortality were 17.10 and 19.61 per 1000 person-years in AGI nonusers and users, respectively. Compared with nonusers, AGI users had a higher mortality risk [adjusted hazard ratio (aHR) = 1.21, 95% confidence interval (CI) = 1.05–1.40; *p* = 0.01]. Regarding AGI use, aHRs (95% CI) for cardiovascular death, non-cardiovascular death, hospitalized CAD, stroke, and heart failure were 1.20 (0.83–1.74), 1.27 (1.07–1.50), 1.12 (0.95–1.31), 0.98 (0.85–1.14), and 1.03 (0.87–1.22) respectively.

**Conclusion:**

AGI use was associated with higher risks of all-cause mortality and non-cardiovascular death in insulin-treated patients with T2DM. Therefore, adding AGIs in insulin-treated patients may not be appropriate.

## Background

Hyperglycemia increases cardiovascular risks [1]; even when lower than the diabetic threshold, blood glucose levels remain a continuous risk factor for cardiovascular death [2]. After adjustments for major risk factors, type 2 diabetes mellitus (T2DM) is associated with considerably increased risks of premature cardiovascular and non-cardiovascular deaths [3]. Thus, in the current clinical scenario, maintaining blood glucose levels within the reference range and preventing diabetic complications are essential. However, T2DM is a progressive disease characterized by a slow, continuous decline in β-cell function [4]. The UK Prospective Diabetes Study (UKPDS) revealed that after 3 years, approximately 50% of the patients required multiple therapies to meet their glycemic target [5]. Moreover, administration of exogenous insulin injections is frequently required for adequately treat T2DM. Even with insulin therapy, approximately 70% of patients with T2DM do not reach their therapeutic goal [6]. Insulin-treated patients may have higher mortality risks than do non–insulin-treated patients [7]. Yki-Järvinen [8] reported that using insulin combination therapy instead of insulin monotherapy can improve glycemic control. However, objective data on the combined use of oral agents and insulin injections are scant, and there is no consensus regarding the optimal insulin combination therapy.

Alpha-glucosidase inhibitors (AGIs) can reversibly bind to the carbohydrate-binding region of alpha-glucosidases and thereby compete with oligosaccharide binding and delay the cleavage of oligosaccharides to monosaccharides. AGIs can thus retard intestinal glucose absorption and blunt postprandial hyperglycemia [9]. The reduction of postprandial glucose levels can attenuate glucose toxicity and increase insulin sensitivity. The results from the acarbose arm of UKPDS revealed that over a 3-year treatment course, acarbose had a persistent hemoglobin A1c (HbA1c) level–reducing effect similar to that of other therapies [10]. AGIs can also reduce fasting blood glucose levels and postprandial glucose excursion without increasing body weight. AGIs could be ideal complementary drugs in insulin combination therapy. Additionally, rice is the staple food in Asian people, AGIs can retard the absorption of carbohydrate and play an important role in controlling blood sugar in this area.

The Acarbose Cardiovascular Evaluation (ACE) trial [11]—a randomized, controlled trial in patients with impaired glucose tolerance and coronary heart disease—reported that acarbose had a neutral effect on major cardiovascular event outcomes. However, several short-term (< 1 year) randomized, controlled trials have demonstrated that acarbose can significantly reduce HbA1c and postprandial glucose levels in insulin-treated patients with T2DM [12, 13]. Furthermore, no relevant long-term results have been reported; therefore, in the current nationwide retrospective cohort study, we evaluated the long-term outcomes of adding AGIs in insulin-treated patients with T2DM.

## Methods

### Data source

National Health Insurance (NHI) is a mandatory, single-payer insurance program, implemented in Taiwan in 1995, that enrolls approximately 99% of Taiwan’s residents (approximately 23 million people) [14]. We retrieved data from the NHI Research Database (NHIRD), which contains beneficiaries’ information, including outpatient and inpatient claims, drug prescriptions, and medical procedures. Longitudinal Health Insurance Database 2000 (LHID2000), an NHIRD dataset, contains all the original claims data of 1,000,000 beneficiaries, randomly sampled from the NHIRD beneficiaries in 2000. These data include beneficiaries’ birthdate, sex, medical orders, procedures, and medical diagnoses [according to the International Classification of Diseases, Ninth Revision, Clinical Modification (ICD-9-CM)]. We collected patient information from the LHID2000. In the LHID2000, information that could be used to identify individuals or care providers is concealed before release to researchers. The Research Ethics Committee of China Medical University and Hospital approved our study (CMUH104-REC2–115) and waived the need for informed consent.

### Study population

The overall observation period of this retrospective cohort study was 14 years (January 1, 2000, to December 31, 2013). We selected patients newly diagnosed as having T2DM (ICD-9-CM: 250.x) at the age of 30–100 years during 2000–2012. To ensure diagnostic accuracy, only patients with ≥2 diagnoses of T2DM in the outpatient claims or ≥ 1 discharge diagnosis of T2DM in the inpatient claims were considered eligible for inclusion. All patients received insulin treatment after T2DM diagnosis.

We excluded patients diagnosed as having type 1 diabetes mellitus (ICD-9-CM: 250.1x) and hepatic failure (ICD-9-CM: 570, 572.2, 572.4, and 572.8), those undergoing dialysis (ICD-9-CM: 39.95, V56.0, V56.8, and V45.1), those with severe hypoglycemia at emergency department visits or hospitalization (to preclude patients with previous hypoglycemia), those with missing basic information, and those who had used AGIs before insulin prescription.

### AGI exposure

We identified patients who received prescriptions of AGIs (including acarbose and miglitol) after receiving insulin therapy. The index date was defined as the date of first AGI use. AGI nonusers, defined as patients who did not use AGIs during the entire observation period, were randomly assigned an index date after insulin therapy, within the follow-up period.

### Comorbidities and other demographic information

The comorbidities included in this study were coronary artery disease (CAD; ICD-9-CM: 410–414), stroke (ICD-9-CM: 430–438), peripheral arterial occlusive disease (ICD-9-CM: 440.2x, 443.9, 84.1x, 39.25, 39.29, 39.50, and 39.59), heart failure (ICD-9-CM: 428), and atrial fibrillation (ICD-9-CM: 427.3). To increase the validity of the comorbidity diagnoses in the administrative dataset, only patients with ≥2 outpatient or ≥ 1 inpatient claim for these comorbidities were included. We also used the Charlson comorbidity index to quantify other comorbidity profiles of the patients [15]; the adapted Diabetes Complications Severity Index (aDCSI) score [16] was used to evaluate diabetes severity. The Charlson comorbidity index and Diabetes Complications Severity Index scores were calculated according to the patient status 1 year before the index date. We considered the use of basal, premixed, and prandial insulin; antidiabetic drugs other than AGIs (including metformin, sulphonylureas, dipeptidyl peptidase-4 inhibitors, and thiazolidinediones) after the date of diagnosis of diabetes; antihypertensive drugs [such as angiotensin-converting enzyme inhibitors (ACEIs), angiotensin II receptor blockers (ARBs), beta-blockers, calcium-channel blockers, diuretics, and potassium-sparing diuretics]; statins; and aspirin.

### Main outcomes

The primary outcome of this study was all-cause mortality. Mortality was defined by discharge from hospital with certified death (the discharge date was defined as the death date) or by termination of the NHI coverage after discharge from hospital due to critical illness (the end of NHI coverage was defined as the death date). The secondary outcomes of this study were CAD, stroke, and heart failure hospitalizations. These cardiovascular events were assessed from the CAD, stroke, or heart failure diagnoses at hospitalization. Diagnostic accuracy of using ICD-9-CM in NHIRD has been validated previously [17]. The observation period was from the index date to the date of withdrawal from NHI, the date the outcomes were first noted, or December 31, 2013 (the end of the study), whichever occurred first. The primary diagnosis at discharge from hospitalization within 3 months before death was assessed for possible identifiable causes of death [18]. Identifiable causes of cardiovascular death were appropriated from the draft definition provided by Hicks et al. [19]; the diagnoses not included in cardiovascular-related deaths were classified as non-cardiovascular deaths.

### Statistical analysis

Propensity score-matching was adopted to minimize bias due to confounding variables and augment comparability between the two study groups [20]. Nearest-neighbor probability was measured using a multivariable logistic regression model with AGI receipt as a dependent variable to construct matched pairs, in which the proportion between 0.995 and 1.0 indicated a perfect analog. We performed 1:1 propensity score-matching for sex, age, Charlson comorbidity index, Diabetes Complications Severity Index score, and comorbidity as well as for use of antidiabetic drug, antihypertensive drug, statin, and aspirin, by using multiple logistic regression analysis. Demographic information was analyzed using the chi-square and Student *t* tests for categorical and continuous variables, respectively. Cox proportional hazard model was applied to estimate hazard ratios (HRs) and their 95% confidence intervals (CIs) for events related to AGI use. The Kaplan–Meier method was used to calculate cumulative incidence of mortality, and the log-rank test was used to compare the difference in mortality between AGI users and nonusers. To evaluate the dose effect, we analyzed the risk of all-cause mortality according to the cumulative defined daily dose (DDD) of AGI use (< 12.47, 12.47–74.96, or > 74.96 DDD/year), relative to AGI nonuse. Here, defined daily dose, the assumed average maintenance dose per day for adults, for both acarbose and miglitol was 300 mg. We considered a two-tailed *p* of < 0.05 to be significant. The SAS (version 9.4 for Windows; SAS Institute, Inc., Cary, NC, USA) was used for data analysis.

## Results

**Fig. 1:**
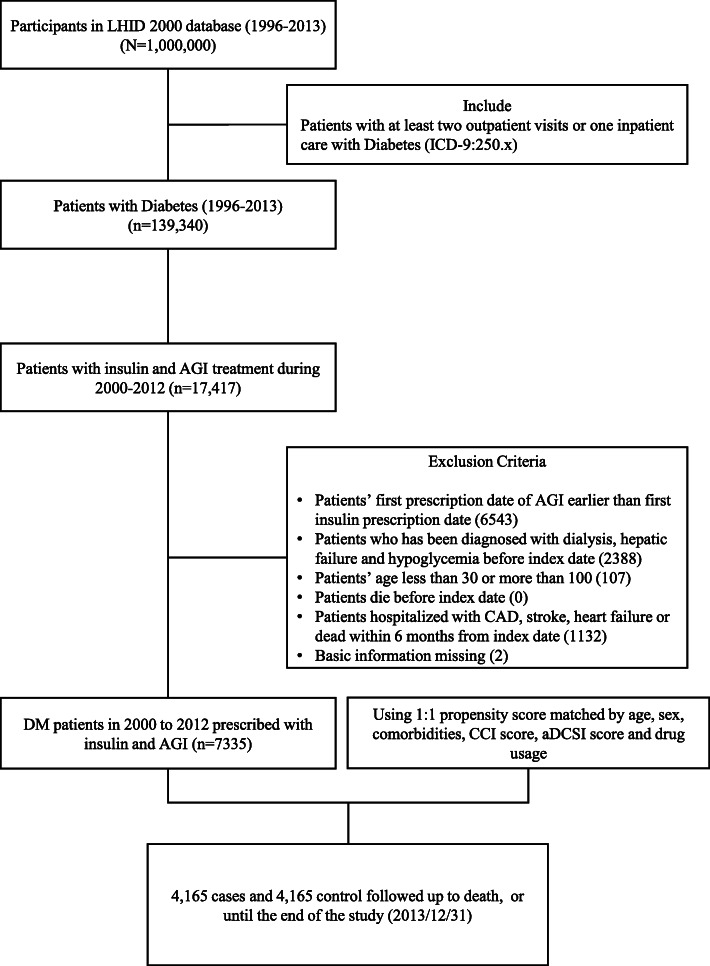
Flow of patient selection and study design

In total, 17,417 patients newly diagnosed as having T2DM receiving insulin therapy during 2000–2012 were included (Fig. 1). After exclusion of ineligible patients, 7335 AGI users and 6683 nonusers remained (Table 1). Compared with AGI users, more AGI nonusers were male and elderly; moreover, the nonusers had shorter diabetes durations, higher Charlson comorbidity index scores, lower Diabetes Complications Severity Index scores, and lower CAD incidence rates and had undergone less intensive treatments. After 1:1 propensity score-matching, the aforementioned variables did not differ significantly between AGI users and nonusers. Finally, both the study (users) and control (nonusers) cohorts each included 4165 patients. Approximately 48.1% of all patients were women. The mean (standard deviation) age of AGI users and non-users were 64.2 (13.1) and 64.3 (13.2) years; the mean follow-up time of AGI users and non-users were 5.38 (3.22) and 4.78 (3.13) years, respectively.

**Table 1:** Baseline characteristics of patients with T2DM receiving insulin with or without AGIs

Variable	Before PSM			***P***-value	After PSM			***P***-value
	DM with insulin treatment				DM with insulin treatment			
	Total ***N*** = 14,018	Non-AGI users (***n*** = 6683)	AGI users (***n*** = 7335)		Total ***N*** = 8330	Non-AGI users (***n*** = 4165)	AGI users (***n*** = 4165)	
	n	n (%) / Mean (SD)	n (%) / Mean (SD)		n	n (%) / Mean (SD)	n (%) / Mean (SD)	
Gender				0.027				0.324
Female	6736	3146 (47.1)	3590 (48.9)		4001	1978 (47.5)	2023 (48.6)	
Male	7282	3537 (52.9)	3745 (51.1)		4329	2187 (52.5)	2142 (51.4)	
Age, years				< 0.001				0.864
< 40	684	356 (5.3)	328 (4.5)		345	177 (4.3)	168 (4.0)	
40–64	6463	2871 (43.0)	3592 (49.0)		3814	1900 (45.6)	1914 (46.0)	
≥ 65	6871	3456 (51.7)	3415 (46.6)		4171	2088 (50.1)	2083 (50.0)	
Mean (SD)		64.7 (14.1)	63.3 (12.9)	< 0.001		64.3 (13.2)	64.2 (13.1)	0.833
Comorbidity								
Coronary artery disease	6334	2895 (43.3)	3439 (46.9)	< 0.001	3773	1887 (45.3)	1886 (45.3)	0.982
Stroke	4609	2233 (33.4)	2376 (32.4)	0.199	2740	1380 (33.1)	1360 (32.7)	0.641
Heart failure	2403	1178 (17.6)	1225 (16.7)	0.146	1391	696 (16.7)	695 (16.7)	0.977
PAOD	1429	629 (9.4)	800 (10.9)	0.004	892	438 (10.5)	454 (10.9)	0.571
Atrial fibrillation	672	358 (5.4)	314 (4.3)	0.003	387	188 (4.5)	199 (4.8)	0.567
CCI score				< 0.001				0.811
0	6281	2809 (42)	3472 (47.3)		3702	1857 (44.6)	1845 (44.3)	
1	2954	1420 (21.2)	1534 (20.9)		1748	862 (20.7)	886 (21.3)	
≧2	4783	2454 (36.7)	2329 (31.8)		2880	1446 (34.7)	1434 (34.4)	
aDCSI score				< 0.001				0.867
0	1903	1065 (15.9)	838 (11.4)		1079	534 (12.8)	545 (13.1)	
1	1268	590 (8.8)	678 (9.2)		725	358 (8.6)	367 (8.8)	
≧2	10,847	5028 (75.2)	5819 (79.3)		6526	3273 (78.6)	3253 (78.1)	
Medications								
Insulin								
Basal insulin	7355	2772 (41.5)	4583 (62.5)	< 0.001	4535	2271 (54.5)	2264 (54.4)	0.878
Premixed insulin	4226	1451 (21.7)	2775 (37.8)	< 0.001	2535	1268 (30.4)	1267 (30.4)	0.981
Prandial insulin	12,763	5966 (89.3)	6797 (92.7)	< 0.001	7602	3805 (91.4)	3797 (91.2)	0.756
Other antidiabetic drugs								
Metformin	11,807	4938 (73.9)	6869 (93.6)	< 0.001	7620	3827 (91.9)	3793 (91.1)	0.182
DPP-4 inhibitor	4825	1403 (21)	3422 (46.7)	< 0.001	2736	1349 (32.4)	1387 (33.3)	0.375
Sulfonylurea	11,536	4848 (72.5)	6688 (91.2)	< 0.001	7408	3719 (89.3)	3689 (88.6)	0.295
TZD	5914	1684 (25.2)	4230 (57.7)	< 0.001	3307	1642 (39.4)	1665 (40)	0.607
Antihypertensive drugs								
ACEI/ARB	11,229	5060 (75.7)	6169 (84.1)	< 0.001	6788	3406 (81.8)	3382 (81.2)	0.498
β-blocker	7654	3462 (51.8)	4192 (57.2)	< 0.001	4392	2204 (52.9)	2188 (52.5)	0.726
Calcium-channel blocker	8078	3683 (55.1)	4395 (59.9)	< 0.001	4690	2353 (56.5)	2337 (56.1)	0.724
Diuretic	8148	3731 (55.8)	4417 (60.2)	< 0.001	4666	2354 (56.5)	2312 (55.5)	0.354
Potassium sparing diuretic	3091	1354 (20.3)	1737 (23.7)	< 0.001	1757	891 (21.4)	866 (20.8)	0.502
Other drugs								
Statin	8013	3134 (46.9)	4879 (66.5)	< 0.001	4877	2443 (58.7)	2434 (58.4)	0.841
Aspirin	8208	3751 (56.1)	4457 (60.8)	< 0.001	4740	2389 (57.4)	2351 (56.4)	0.401
DM duration, year		12.1 (4.5)	12.8 (4.5)	< 0.001		12.3 (4.4)	12.3 (4.7)	0.981

*SD* Standard deviation, *PAOD* Peripheral arterial occlusive disease, *CCI* Charlson comorbidity index, *aDCSI* Adapted Diabetes Complications Severity Index, *DPP-4* dipeptidyl peptidase-4, *TZD* Thiazolidinedione, *ACEI* Angiotensin-converting enzyme inhibitor, *ARB* Angiotensin II receptor blocker

**Fig. 2:**
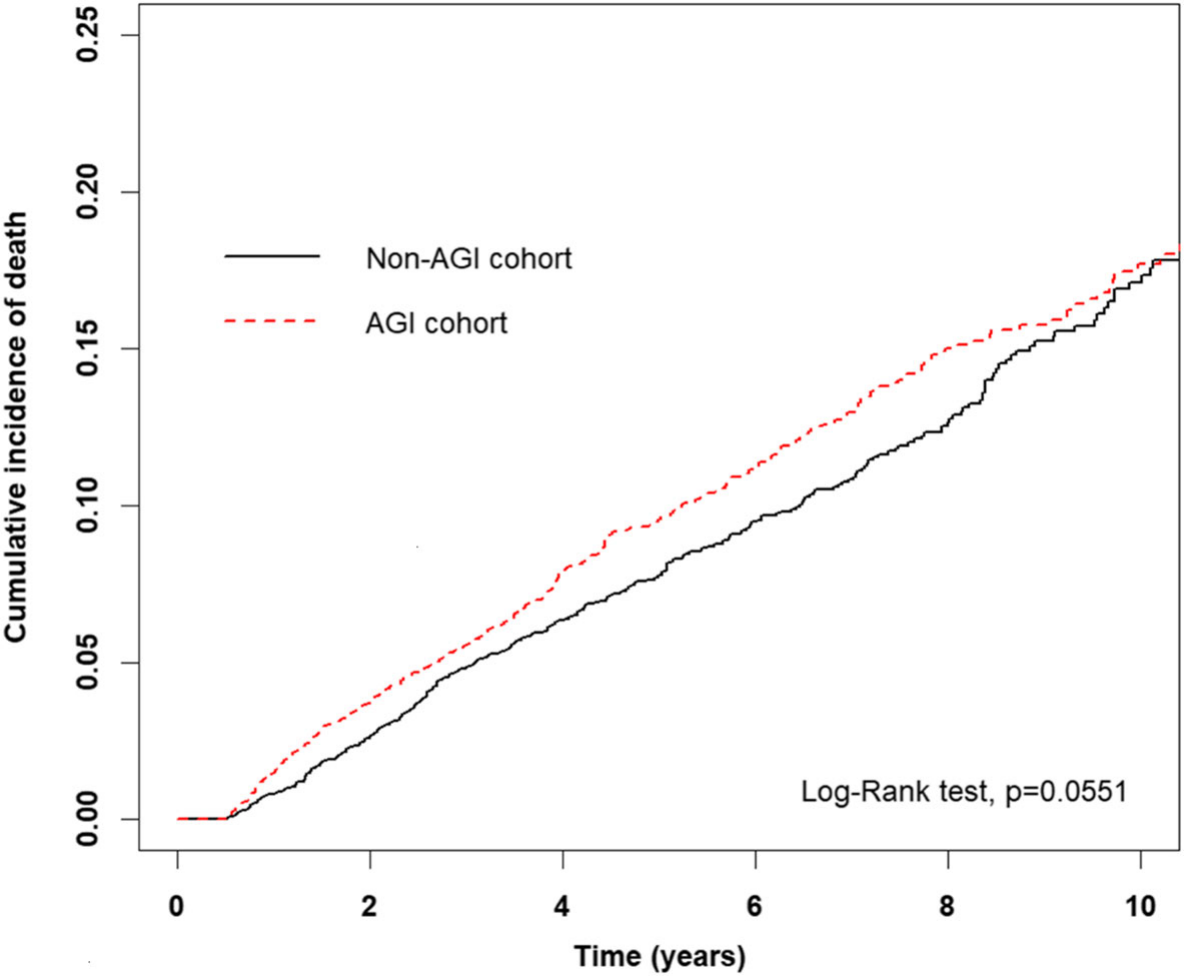
Cumulative incidence of all-cause mortality in AGI users and nonusers

Moreover, in AGI users and nonusers, 405 and 351 deaths occurred, respectively (incidence rate = 19.61 and 17.10 per 1000 person-years, respectively; adjusted HR (aHR) = 1.21, 95% CI = 1.05–1.40; *p* = 0.010; Table 2). Figure 2 illustrates the cumulative incidence of all-cause mortality based on the Kaplan–Meier method results, revealing a higher mortality risk in AGI users than in nonusers (log-rank test, *p* = 0.0551).

**Table 2:** Mortality risk in patients with T2DM receiving insulin treatment

Characteristics	Event no. (***n*** = 756)	Person-Year	IR	Crude		Adjusted	
				HR (95% CI)	***p*** value	HR (95% CI)	***P*** value
AGI users							
No	351	20,529	17.10	Ref.		Ref.	
Yes	405	20,654	19.61	1.15 (1.00–1.33)	0.055	1.21 (1.05–1.40)	0.010
Cumulative defined daily doses of AGI therapy, per year							
Non-users	351	20,529	17.10	Ref.		Ref.	
≦12.47	153	7129	21.46	1.26 (1.04–1.52)	0.018	1.15 (0.95–1.40)	0.141
12.47–74.96	134	7539	17.77	1.04 (0.85–1.26)	0.730	1.15 (0.94–1.40)	0.180
> 74.96	118	5986	17.92	1.17 (0.95–1.44)	0.141	1.41 (1.14–1.74)	0.001
*P* for trend					0.191		0.002
Gender							
Female	318	20,740	15.33	Ref.		Ref.	
Male	438	20,442	21.43	1.41 (1.22–1.63)	< 0.001	1.57 (1.35–1.82)	< 0.001
Age, years							
< 40	6	2072	2.90	Ref.		Ref.	
40–64	195	20,794	9.38	3.31 (1.47–7.47)	0.004	3.23 (1.42–7.31)	0.005
≥ 65	555	18,316	30.30	11.22 (5.02–25.10)	< 0.001	7.25 (3.19–16.45)	< 0.001
Comorbidity							
Coronary artery disease	414	18,013	22.98	1.57 (1.36–1.81)	< 0.001	0.86 (0.73–1.01)	0.074
Stroke	376	12,076	31.13	2.44 (2.12–2.82)	< 0.001	1.3 (1.10–1.52)	0.002
Heart failure	204	5645	36.14	2.40 (2.04–2.82)	< 0.001	1.31 (1.09–1.58)	0.004
PAOD	89	3918	22.72	1.29 (1.03–1.61)	0.026	0.91 (0.72–1.14)	0.401
Atrial fibrillation	67	1432	46.80	2.80 (2.18–3.60)	< 0.001	1.19 (0.91–1.56)	0.201
CCI score							
0	163	19,848	8.21	Ref.		Ref.	
1	154	8752	17.60	2.16 (1.74–2.70)	< 0.001	1.61 (1.28–2.02)	< 0.001
≧2	439	12,583	34.89	4.36 (3.64–5.22)	< 0.001	2.41 (1.97–2.96)	< 0.001
aDCSI score							
0	63	6103	10.32	Ref.		Ref.	
1	44	4188	10.51	1.02 (0.69–1.49)	0.931	1.07 (0.73–1.58)	0.722
≧2	649	30,892	21.01	2.08 (1.61–2.70)	< 0.001	0.92 (0.69–1.23)	0.594
Medications							
Other antidiabetic drugs							
Metformin	671	37,840	17.73	0.69 (0.55–0.87)	0.002	0.79 (0.60–1.03)	0.081
DPP-4 inhibitor	65	14,417	4.51	0.17 (0.13–0.22)	< 0.001	0.25 (0.19–0.33)	< 0.001
Sulfonylurea	667	36,947	18.05	0.86 (0.69–1.07)	0.167	0.93 (0.71–1.21)	0.595
TZD	207	17,940	11.54	0.48 (0.41–0.57)	< 0.001	0.64 (0.54–0.76)	< 0.001
Anti-hypertensive drugs							
ACEI/ARB	648	34,009	19.05	1.26 (1.03–1.55)	0.026	0.78 (0.63–0.97)	0.029
β-blocker	469	23,231	20.19	1.25 (1.08–1.45)	0.003	0.83 (0.70–0.99)	0.041
Calcium-channel blocker	501	24,484	20.46	1.33 (1.14–1.54)	< 0.001	1.05 (0.87–1.26)	0.613
Diuretic	564	23,882	23.62	2.12 (1.80–2.49)	< 0.001	1.43 (1.18–1.74)	< 0.001
Potassium sparing diuretic	295	8435	34.97	2.50 (2.16–2.89)	< 0.001	1.51 (1.29–1.77)	< 0.001
Other drugs							
Statin	352	25,739	13.68	0.51 (0.44–0.59)	< 0.001	0.69 (0.59–0.80)	< 0.001
Aspirin	523	24,954	20.96	1.44 (1.24–1.68)	< 0.001	1.15 (0.95–1.39)	0.140

Adjusted HR was adjusted for sex, age, all comorbidities, and medications in Cox proportional hazards regression

*SD* Standard deviation, *PAOD* Peripheral arterial occlusive disease, *CCI* Charlson comorbidity index, *aDCSI* Adapted Diabetes Complications Severity Index, *DPP-4* Dipeptidyl peptidase-4, *TZD* Thiazolidinedione, *ACEI* Angiotensin-converting enzyme inhibitor, *ARB* Angiotensin II receptor blocker

As presented in Table 2, the aHRs of < 12.47, 12.47–74.96, and > 74.96 cumulative DDD/year of AGI for all-cause mortality were 1.15, 1.15, and 1.41, respectively, with the *p* for trend = 0.002. As presented in Table 3, the aHRs for cardiovascular death, non-cardiovascular death, hospitalized CAD, stroke, and heart failure in AGI users compared with nonusers were 1.20, 1.27, 1.12, 0.98, and 1.03, respectively.

**Table 3:** IRs and HRs for mortality, CAD, stroke, and HF hospitalization

Outcomes	AGI						Case vs. control	
	No			Yes			Crude HR	Adjusted HR
	Event	Person Years	IR	Event	Person Years	IR	(95% CI)	(95% CI)
CV death	54	20,529	2.63	59	20,654	2.86	1.10 (0.76–1.60)	1.20 (0.83–1.74)
Non-CV death	259	20,529	12.62	314	20,654	15.20	1.21 (1.02–1.42)^*^	1.27 (1.07–1.50)^**^
Hospitalized CAD	295	20,511	14.38	332	20,563	16.15	1.12 (0.96–1.31)	1.12 (0.95–1.31)
Hospitalized stroke	358	20,332	17.61	354	20,533	17.24	0.98 (0.85–1.13)	0.98 (0.85–1.14)
Hospitalized HF	281	20,042	14.02	288	20,081	14.34	1.02 (0.87–1.21)	1.03 (0.87–1.22)

Adjusted HR was adjusted for sex, age, urbanization, and all comorbidities in Cox proportional hazards regression

*IR* Incidence rate (per 1000 person-years), *HR* Hazard ratio, *CI* Confidence interval, *CV* Cardiovascular death, *CAD* Coronary artery disease, *HF* Heart failure

^*^p < 0.05, ^**^p < 0.01

Factors that increased risk of all-cause mortality included Charlson comorbidity index score of 1 or ≥ 2, stroke or heart failure history, and diuretics use. Male patients as well as those aged 40–64 years or ≥ 65 years were also at higher risk of all-cause mortality (Table 2). Dipeptidyl peptidase-4 inhibitor, thiazolidinedione, ACEI or ARB, beta-blocker, and statin use was associated with lower mortality risks.

## Discussion

After propensity score-matching for multiple clinical variables, adding AGIs in insulin-treated patients were associated with higher risks of all-cause mortality compared with insulin-treated patients not adding AGIs. This mortality risk associated with AGI use displayed a dose–response trend. The increased risk of mortality was predominantly associated with non-cardiovascular death. Our study also demonstrated that adding AGIs in insulin-treated patients had no significant effect on the risks of hospitalized CAD, stroke, and heart failure as compared with patients not adding AGIs.

The STOP-NIDDM trial results revealed that acarbose use in patients with impaired glucose tolerance reduced cardiovascular disease risk [21]. However, the ACE trial indicated that in Chinese patients with impaired glucose tolerance and coronary heart disease, acarbose did not reduce the risk of major cardiovascular events [11]. A meta-analysis of seven long-term randomized controlled trials found that compared with a placebo, acarbose could reduce myocardial infarction and cardiovascular event risks in patients with T2DM [22]. In their nationwide cohort study, Chang et al. demonstrated that metformin add-on AGI was associated with lower acute myocardial infarction risks compared with metformin add-on sulphonylurea [23]. Hsu et al. compared the use of acarbose as an add-on treatment to metformin with sulfonylurea, and reported low myocardial infarction and ischemic stroke hospitalization risks, but no effect on all-cause mortality [24]. AGIs can reduce HbA1c, postprandial glucose levels, and glucose excursion [25]. Subsequently, reduction in blood glucose levels can diminish the superoxide overproduction in the mitochondrial electron transport chain, which reduces oxidative stress [25]. Some studies have also determined that AGI use can restore endothelial function and reduce carotid intima–media thickening, low-grade inflammation, and cardiac interstitial fibrosis [25]. However, the Cochrane systematic review of acarbose use in patients with T2DM did not reveal any prognostic benefits on mortality or morbidity [26]. The long-term outcomes of AGI use remain inconclusive.

Some randomized controlled trials of adding acarbose in insulin-treated patients have revealed significant reductions in HbA1c and postprandial glucose levels [12, 13]; however, long-term outcomes have not been reported. The present study demonstrated that adding AGIs in insulin-treated patients have no significant effect on major cardiovascular events and cardiovascular death. This result is consistent with that of the aforementioned Cochrane systematic review [26] but differs from that of the MeRia study, Chang et al., and Hsu et al. This difference may be due to our patients having received insulin therapy; whereas in the other studies, patients were administered AGI monotherapy or metformin add-on dual therapy. Insulin-treated patients had longer T2DM duration with more complications, and AGI administration may not have been sufficient to reverse the atherosclerotic events.

Our study indicated that AGI use in insulin-treated patients may increase mortality risk and that higher cumulative defined daily dose of AGI can increase the propensity of mortality compared with AGI nonuse. The mechanism of action of AGI in insulin-treated patients and the resulting mortality risk remains unknown. AGI use does not appear to have severe toxicity, except for flatulence and diarrhea, which are commonly noted among AGI users [9]. Poor appetite with flatulence and dehydration after diarrhea should be closely monitored in AGI users to prevent the related mortality.

Taken together, the following are our speculations regarding mortality risk and adding AGIs in insulin-treated patients with T2DM. Although we balanced basal, prandial insulin, and oral antidiabetic drugs use between the study and control cohorts, combining AGI and insulin to treat hyperglycemia may not be as efficient for glycemic control as increasing insulin or combining insulin with other antidiabetic agents; the mortality risk reduction may also not be as efficient as other antidiabetic agents. Another possibility is that individuals who were on larger cumulative defined daily doses of AGI might have more suboptimal glycemic control with possible higher complication burden and mortality risk, and therefore required higher dose of AGI for achieving optimal glycemic control.

According to the draft of definition for cardiovascular-related causes of death by Hicks et al. [19], which does not include cerebrovascular diseases, the proportion of cardiovascular and non-cardiovascular deaths in our study were 14.9 and 75.8%, respectively. This result is consistent with the report of the national survey of mortality in Taiwan’s patients with T2DM during 2005–2014 [27]. Thus, most of the deaths in our insulin-treated patients with T2DM were non-cardiovascular.

Men, older patients, and patients with further comorbidities presented a higher mortality risk; these are high-risk groups who need closely monitoring when considering insulin treatments. This study demonstrated that statin use was associated with a lower mortality risk than was statin nonuse—consistent with a previous meta-analysis of statin therapy [28]. Dipeptidyl peptidase-4 inhibitor use in insulin-treated patients was associated with higher survival rates—consistent with our previous results [29]. Thus, the addition of dipeptidyl peptidase-4 inhibitors to insulin treatment may be a promising approach.

Our study revealed that ACEI and ARB use reduced mortality risk in insulin-treated patients; however, diuretic use for hypertensive treatment increased mortality risk. Most data suggested that adequate blood pressure control may be more critical than selecting particular agents for the management of hypertension [30]. As per the American Diabetes Association guidelines [30], the treatment of hypertension in patients with diabetes may include drugs that reduce the risk of cardiovascular events (such as ACEI, ARB or diuretics). However, the studies on the optimal drug choice for hypertensive treatment in patients with underlying insulin treatments are limited. Our study revealed that for treating hypertension in insulin-treated patients, ACEI or ARB might be more adequate than diuretics.

There are some limitations in this study. First, information on patients’ smoking status, alcohol consumption, physical activity level, body weight, and height are not provided in the NHIRD; similarly, blood glucose, HbA1c, cholesterol levels and renal function status are not available in the database. Nevertheless, through the propensity score-matching of multiple critical variables by maximally controlling for the confounding factors has potentially increased the accuracy of our results. Second, when using the claims data for analysis, assessing patient adherence to the dosage of prescribed insulin or oral antidiabetic drugs is difficult. In addition, as insulin prescription in the NHI system is monitored as the number of insulin pens used for reimbursement purposes, we could not derive an accurate insulin dosage, which may influence the results of mortality and cardiovascular events. Third, we did not measure the risk of hypoglycemia in our study because we could not calculate the rates for hypoglycemia accurately from the database. Hypoglycemia incidence in outpatient claims might be underestimated because patients do not report their hypoglycemia to their physicians; inpatient records of hypoglycemia might also be underestimated because some hypoglycemic patients are cared for by family members without being admitted to hospitals or are referred to the emergency department without admission. Finally, because this was a retrospective cohort study, it contained some inevitable biases; a large scale randomized trial is needed to evaluate our results for therapeutic applications.

## Conclusions

In summary, adding AGIs in insulin-treated patients might increase all-cause mortality risk as compared with no adding AGIs; and most of the increased death risk is from non-cardiovascular causes. Thus, future studies on addition of antidiabetic agents other than AGIs with insulin for superior glycemic control and long-term favorable prognosis are warranted.

## Data Availability

Data are available from the National Health Insurance Research Database (NHIRD) published by Taiwan National Health Insurance (NHI) Bureau. The data utilized in this study cannot be made available in the paper, the supplemental files, or in a public repository due to the “Personal Information Protection Act” executed by Taiwan’s government, starting from 2012. Requests for data can be sent as a formal proposal to the NHIRD (http://nhird.nhri.org.tw) or by email to nhird@nhri.org.tw.

## Abbreviations

T2DM: Type 2 diabetes mellitus
AGI: Alpha-glucosidase inhibitor
CAD: Coronary artery disease
DDD: Defined daily dose
